# 2-[(*E*)-2-(1*H*-Indol-3-yl)ethen­yl]-1-methyl­pyridinium 4-bromo­benzene­sulfonate^1^

**DOI:** 10.1107/S1600536809011386

**Published:** 2009-04-02

**Authors:** Suchada Chantrapromma, Thawanrat Kobkeatthawin, Hoong-Kun Fun

**Affiliations:** a Crystal Materials Research Unit, Department of Chemistry, Faculty of Science, Prince of Songkla University, Hat-Yai, Songkhla 90112, Thailand; b X-ray Crystallography Unit, School of Physics, Universiti Sains Malaysia, 11800 USM, Penang, Malaysia

## Abstract

In the title compound, C_16_H_15_N_2_^+^·C_6_H_4_BrO_3_S^−^, the cation exists in the *E* configuration and is essentially planar with a dihedral angle of 3.10 (5)° between the pyridinium ring and the indole ring system. The π-conjugated planes of the cation and the anion are inclined to each other at a dihedral angle of 64.32 (4)°. In the crystal structure, the cations are stacked in an anti­parallel manner along the *a* axis. The anions are linked into a chain along the *a* axis. The cations and the anions are linked into a three-dimensional network by N—H⋯O and weak C—H⋯O hydrogen bonds. The crystal structure is further stabilized by C—H⋯π inter­actions. A π–π inter­action between the five-membered heterocyclic ring of the indole system and the pyridinium ring is also observed with a centroid–centroid distance of 3.5855 (7) Å.

## Related literature

For bond-length data, see: Allen *et al.* (1987 ▶). For background to non-linear optical materials research, see: Coe *et al.* (2003 ▶); Dittrich *et al.* (2003 ▶); Ogawa *et al.* (2008 ▶); Otero *et al.* (2002 ▶); Weir *et al.* (2003 ▶); Yang *et al.* (2007 ▶). For related structures, see, for example: Chanawanno *et al.* (2008 ▶); Chantrapromma *et al.* (2006 ▶, 2007 ▶, 2008 ▶, 2009 ▶); Jindawong *et al.* (2005 ▶). For the stability of the temperature controller used in the data collection, see: Cosier & Glazer (1986 ▶).
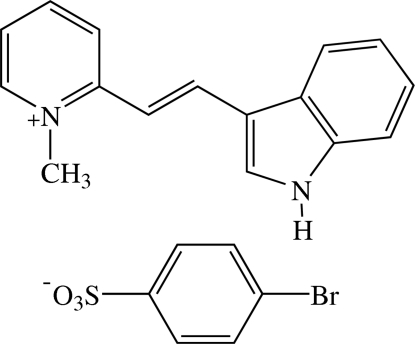


## Experimental

### 

#### Crystal data

C_16_H_15_N_2_^+^·C_6_H_4_BrO_3_S^−^
*M* *_r_* = 471.36
Monoclinic, 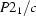

*a* = 7.5188 (1) Å
*b* = 13.3659 (2) Å
*c* = 20.2670 (3) Å
β = 98.850 (1)°
*V* = 2012.49 (5) Å^3^
*Z* = 4
Mo *K*α radiation
μ = 2.17 mm^−1^
*T* = 100 K
0.31 × 0.27 × 0.16 mm

#### Data collection

Bruker APEXII CCD area-detector diffractometer
Absorption correction: multi-scan (**SADABS**; Bruker, 2005 ▶) *T*_min_ = 0.548, *T*_max_ = 0.706
67716 measured reflections
10581 independent reflections
8073 reflections with *I* > 2σ(*I*)
*R*_int_ = 0.033

#### Refinement

*R*[*F* ^2^ > 2σ(*F* ^2^)] = 0.033
*wR*(*F* ^2^) = 0.085
*S* = 1.03
10581 reflections
267 parameters
H atoms treated by a mixture of independent and constrained refinement
Δρ_max_ = 1.02 e Å^−3^
Δρ_min_ = −0.66 e Å^−3^

### 

Data collection: *APEX2* (Bruker, 2005 ▶); cell refinement: *SAINT* (Bruker, 2005 ▶); data reduction: *SAINT*; program(s) used to solve structure: *SHELXTL* (Sheldrick, 2008 ▶); program(s) used to refine structure: *SHELXTL*; molecular graphics: *SHELXTL*; software used to prepare material for publication: *SHELXTL* and *PLATON* (Spek, 2009 ▶).

## Supplementary Material

Crystal structure: contains datablocks global, I. DOI: 10.1107/S1600536809011386/is2401sup1.cif

Structure factors: contains datablocks I. DOI: 10.1107/S1600536809011386/is2401Isup2.hkl

Additional supplementary materials:  crystallographic information; 3D view; checkCIF report

## Figures and Tables

**Table 1:** Hydrogen-bond geometry (Å, °)

*D*—H⋯*A*	*D*—H	H⋯*A*	*D*⋯*A*	*D*—H⋯*A*
N2—H1*N*2⋯O2^i^	0.85 (2)	1.91 (2)	2.7593 (14)	175.2 (17)
C1—H1*A*⋯O3^ii^	0.93	2.53	3.2067 (16)	130
C7—H7*A*⋯O1	0.93	2.58	3.3095 (16)	136
C9—H9*A*⋯O1	0.93	2.58	3.2426 (16)	128
C14—H14*A*⋯O1^iii^	0.93	2.56	3.2987 (16)	137
C16—H16*C*⋯O1^iii^	0.96	2.36	3.2739 (17)	158
C19—H19*A*⋯O3^iv^	0.93	2.51	3.2052 (16)	131
C21—H21*A*⋯O2^v^	0.93	2.28	3.1310 (15)	152
C4—H4*A*⋯*Cg*3	0.93	2.82	3.5579 (13)	137
C16—H16*A*⋯*Cg*3^vi^	0.96	2.69	3.5731 (13)	154
C16—H16*B*⋯*Cg*1^vii^	0.96	2.74	3.4836 (14)	135

Symmetry codes: (i) 
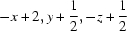
; (ii) 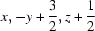; (iii) 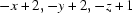; (iv) 
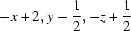
; (v) 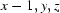; (vi) 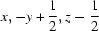; (vii) 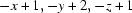. *Cg*1 and *Cg*3 are the centroids of the N2/C8–C10/C15 and C10–C15 rings, respectively.

**Fig. 1.**
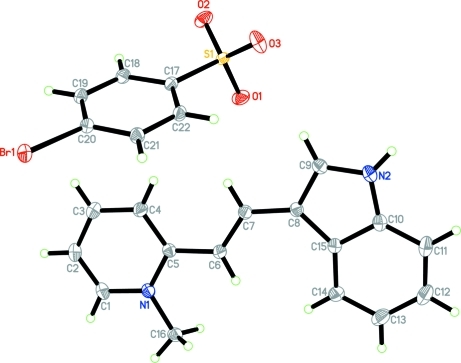
The molecular structure of the title compound, with 50% probability displacement ellipsoids and the atom-numbering scheme.

**Fig. 2.**
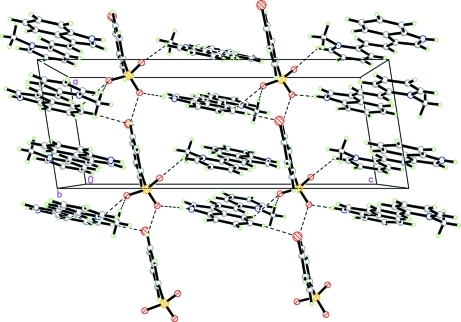
The crystal packing of the title compound, viewed down the b axis. Hydrogen bonds are shown as dashed lines.

## supplementary crystallographic information

### Comment

Organic crystals with extensive conjugated π systems are attractive candidates for nonlinear optic (NLO) studies because of their large hyperpolariability (β) and ease of preparation (Coe *et al.*, 2003; Dittrich *et al.*, 2003; Ogawa *et al.*, 2008; Otero *et al.*, 2002; Weir *et al.*, 2003; Yang *et al*., 2007). One strategy to enhance the hyperpolariability of the cations is by elongation of its π-conjugation system. Based on these studies, we have previously synthesized and reported the crystal structure of the pyridinium salts (Chanawanno *et al.*, 2008; Chantrapromma *et al.*, 2006, 2007, 2008, 2009; Jindawong *et al.*, 2005) in order to study for their NLO properties. We herein report the crystal structure of the title compound, (I), which is another pyridinium salt.

Figure 1 shows the asymmetric unit of (I), which consists of a C_16_H_15_N_2_^+^ cation and a C_6_H_4_BrO_3_S^-^ anion. The cation exists in the *E* configuration with respect to the C6═C7 double bond [1.3568 (16) Å] and is essentially planar with a dihedral angle between the pyridinium and indole rings being 3.10 (5)°, the torsion angles C4–C5–C6–C7 = -2.13 (19)° and C6–C7–C8–C15 = 3.9 (2)°. The indole ring system is planar with the most deviation of -0.0137 (12) Å for atom C8. The π-conjugated planes of the cation and the anion are inclined to each other with the interplanar angle between them being 64.32 (4)°. The methyl group is co-planar with the attached N1/C1–C5 ring. The bond lengths in (I) are in normal ranges (Allen *et al.*, 1987) and comparable with those in related structures (Chanawanno *et al.*, 2008; Chantrapromma *et al.*, 2006, 2007, 2008, 2009; Jindawong *et al.*, 2005).

In the crystal packing (Fig. 2), all O atoms of the sulfonate group are involved in weak C—H···O interactions (Table 1). The arrangement of the cations and anions is interesting (Fig. 2). The cations are stacked in an antiparallel manner along the *a* axis and the anions are linked together into chains along the same direction. The cations are linked to the anions into a three dimensional network by N—H···O hydrogen bonds and weak C—H···O interactions (Table 1). The crystal structure is further stabilized by C—H···π interactions (Table 1). A π–π interaction with a distance *Cg*1···*Cg*2 = 3.5855 (7) Å (symmetry code: 2-x, 2-y, 1-z) is observed; *Cg*1 and *Cg*2 are the centroids of the N2/C8–C10/C15 and N1/C1–C5 rings, respectively.

### Experimental

A solution of indole-3-carboxaldehyde (2.47 g, 17.02 mmol) in methanol (25 ml) was added dropwise to a stirred solution of 1,2-dimethylpyridinium iodide (4.00 g, 17.02 mmol) in methanol (15 ml) in the presence of piperidine (1.68 ml, 17.02 mmol) over a period of 15 mins at room temperature. The mixture was then refluxed for 1 hr in the nitrogen atmosphere. The solid formed was filtered, washed with diethyl ether and recrystallized from methanol to give orange crystals of 2-[(*E*)-2-(1*H*-Indol-3-yl) ethenyl]-1-methylpyridinium iodide (compound A) (5.61 g, 91%; m.p. 537-539 K).

Silver(I) *p*-bromobenzenesulfonate (compound B) was synthesized according to our previously reported procedure (Chantrapromma *et al.*, 2006). The title compound was synthesized by disolving compound B (0.20 g, 0.58 mmol) in 20 ml methanol which upon heating was added a solution of compound A (0.21 g, 0.58 mmol) in hot methanol (30 ml). The mixture turned yellow and cloudy immediately. After stirring for 0.5 hr, the precipitate of silver iodide was filtered and the filtrate was evaporated to give an orange gum. Orange block-shaped single crystals of the title compound suitable for *x*-ray structure determination were recrystalized from methanol by slow evaporation of the solvent at room temperature after a few weeks (m.p. 508-510 K).

### Refinement

H atom attached to N was located from the difference map and refined isotropically. The remaining H atoms were placed in calculated positions with C—H = 0.93 Å and *U*_iso_(H) = 1.2*U*_eq_(C) for aromatic and CH, and with C—H = 0.96 Å and *U*_iso_(H) = 1.5*U*_eq_(C) for CH_3_ atoms. A rotating group model was used for the methyl groups. The highest residual electron density peak is located at 0.59 Å from S1 and the deepest hole is located at 0.36 Å from Br1.

### Crystal data

**Table:**

C_16_H_15_N_2_^+^·C_6_H_4_BrO_3_S^−^	*F*(000) = 960
*M**_r_* = 471.36	*D*_x_ = 1.556 Mg m^−^^3^
Monoclinic, *P*2_1_/*c*	Melting point = 508–510 K
Hall symbol: -P 2ybc	Mo *K*α radiation, λ = 0.71073 Å
*a* = 7.5188 (1) Å	Cell parameters from 10581 reflections
*b* = 13.3659 (2) Å	θ = 2.0–37.5°
*c* = 20.2670 (3) Å	µ = 2.17 mm^−^^1^
β = 98.850 (1)°	*T* = 100 K
*V* = 2012.49 (5) Å^3^	Block, yellow
*Z* = 4	0.31 × 0.27 × 0.16 mm

### Data collection

**Table:**

Bruker APEXII CCD area-detector diffractometer	10581 independent reflections
Radiation source: sealed tube	8073 reflections with *I* > 2σ(*I*)
graphite	*R*_int_ = 0.033
φ and ω scans	θ_max_ = 37.5°, θ_min_ = 2.0°
Absorption correction: multi-scan (*SADABS*; Bruker, 2005)	*h* = −11→12
*T*_min_ = 0.548, *T*_max_ = 0.706	*k* = −22→22
67716 measured reflections	*l* = −34→34

### Refinement

**Table:**

Refinement on *F*^2^	Primary atom site location: structure-invariant direct methods
Least-squares matrix: full	Secondary atom site location: difference Fourier map
*R*[*F*^2^ > 2σ(*F*^2^)] = 0.033	Hydrogen site location: inferred from neighbouring sites
*wR*(*F*^2^) = 0.085	H atoms treated by a mixture of independent and constrained refinement
*S* = 1.03	*w* = 1/[σ^2^(*F*_o_^2^) + (0.0369*P*)^2^ + 0.9196*P*] where *P* = (*F*_o_^2^ + 2*F*_c_^2^)/3
10581 reflections	(Δ/σ)_max_ = 0.003
267 parameters	Δρ_max_ = 1.02 e Å^−^^3^
0 restraints	Δρ_min_ = −0.66 e Å^−^^3^

### Special details

**Table:**

Experimental. The crystal was placed in the cold stream of an Oxford Cryosystems Cobra open-flow nitrogen cryostat [Cosier, J. & Glazer, A. M. (1986). *J. Appl. Cryst*. 19, 105–107.] operating at 100.0 (1) K.
Geometry. All esds (except the esd in the dihedral angle between two l.s. planes) are estimated using the full covariance matrix. The cell esds are taken into account individually in the estimation of esds in distances, angles and torsion angles; correlations between esds in cell parameters are only used when they are defined by crystal symmetry. An approximate (isotropic) treatment of cell esds is used for estimating esds involving l.s. planes.
Refinement. Refinement of F^2^ against ALL reflections. The weighted R-factor wR and goodness of fit S are based on F^2^, conventional R-factors R are based on F, with F set to zero for negative F^2^. The threshold expression of F^2^ > 2sigma(F^2^) is used only for calculating R-factors(gt) etc. and is not relevant to the choice of reflections for refinement. R-factors based on F^2^ are statistically about twice as large as those based on F, and R- factors based on ALL data will be even larger.

### Fractional atomic coordinates and isotropic or equivalent isotropic displacement parameters (Å^2^)

**Table:**

	*x*	*y*	*z*	*U*_iso_*/*U*_eq_	
Br1	0.437510 (18)	0.545613 (9)	0.317640 (6)	0.02094 (4)	
S1	1.03248 (4)	0.87587 (2)	0.276111 (13)	0.01297 (5)	
O1	1.08595 (14)	0.92518 (8)	0.33975 (5)	0.02417 (19)	
O2	1.17976 (12)	0.82343 (7)	0.25255 (5)	0.02343 (19)	
O3	0.93322 (13)	0.94158 (8)	0.22583 (5)	0.0253 (2)	
C17	0.87426 (15)	0.78270 (8)	0.29047 (5)	0.01413 (18)	
C18	0.92454 (16)	0.68277 (9)	0.30078 (6)	0.01618 (19)	
H18A	1.0438	0.6639	0.3012	0.019*	
C19	0.79634 (16)	0.61112 (9)	0.31052 (6)	0.0171 (2)	
H19A	0.8285	0.5442	0.3170	0.021*	
C20	0.61934 (16)	0.64165 (8)	0.31029 (6)	0.01558 (19)	
C21	0.56765 (15)	0.74130 (9)	0.30144 (6)	0.01596 (19)	
H21A	0.4491	0.7603	0.3023	0.019*	
C22	0.69622 (15)	0.81182 (8)	0.29127 (6)	0.01531 (18)	
H22A	0.6637	0.8787	0.2850	0.018*	
N1	0.75652 (13)	0.79242 (8)	0.58104 (5)	0.01574 (17)	
N2	0.77966 (15)	1.20325 (8)	0.35483 (5)	0.01918 (19)	
C1	0.77944 (17)	0.69502 (9)	0.59983 (6)	0.0196 (2)	
H1A	0.7581	0.6761	0.6421	0.024*	
C2	0.83291 (18)	0.62419 (9)	0.55835 (7)	0.0218 (2)	
H2A	0.8490	0.5580	0.5721	0.026*	
C3	0.86283 (18)	0.65363 (9)	0.49483 (6)	0.0210 (2)	
H3A	0.8972	0.6067	0.4653	0.025*	
C4	0.84120 (17)	0.75234 (9)	0.47621 (6)	0.0191 (2)	
H4A	0.8621	0.7717	0.4340	0.023*	
C5	0.78805 (15)	0.82472 (9)	0.51967 (5)	0.01522 (18)	
C6	0.76564 (17)	0.92976 (9)	0.50349 (6)	0.01701 (19)	
H6A	0.7261	0.9722	0.5346	0.020*	
C7	0.79955 (16)	0.96932 (9)	0.44504 (6)	0.01598 (19)	
H7A	0.8379	0.9250	0.4148	0.019*	
C8	0.78260 (15)	1.07202 (9)	0.42496 (5)	0.01529 (18)	
C9	0.80825 (16)	1.10341 (9)	0.36157 (6)	0.0178 (2)	
H9A	0.8404	1.0618	0.3286	0.021*	
C10	0.73615 (16)	1.24110 (9)	0.41377 (6)	0.0181 (2)	
C11	0.69668 (18)	1.33969 (10)	0.42910 (7)	0.0234 (2)	
H11A	0.6958	1.3909	0.3980	0.028*	
C12	0.65886 (19)	1.35765 (10)	0.49284 (8)	0.0270 (3)	
H12A	0.6305	1.4223	0.5047	0.032*	
C13	0.66243 (19)	1.28055 (11)	0.53972 (7)	0.0263 (3)	
H13A	0.6384	1.2953	0.5823	0.032*	
C14	0.70096 (18)	1.18259 (10)	0.52424 (6)	0.0210 (2)	
H14A	0.7027	1.1321	0.5559	0.025*	
C15	0.73730 (16)	1.16115 (9)	0.45962 (6)	0.01610 (19)	
C16	0.69856 (17)	0.86295 (10)	0.62973 (6)	0.0191 (2)	
H16A	0.6809	0.8272	0.6693	0.029*	
H16B	0.5877	0.8943	0.6105	0.029*	
H16C	0.7894	0.9132	0.6410	0.029*	
H1N2	0.796 (2)	1.2377 (15)	0.3209 (10)	0.028 (5)*	

### Atomic displacement parameters (Å^2^)

**Table:**

	*U*^11^	*U*^22^	*U*^33^	*U*^12^	*U*^13^	*U*^23^
Br1	0.02422 (6)	0.01477 (5)	0.02501 (6)	−0.00464 (4)	0.00755 (4)	−0.00167 (4)
S1	0.01221 (11)	0.01335 (10)	0.01371 (10)	−0.00058 (8)	0.00315 (8)	−0.00260 (9)
O1	0.0282 (5)	0.0241 (4)	0.0212 (4)	−0.0076 (4)	0.0070 (3)	−0.0102 (4)
O2	0.0167 (4)	0.0236 (4)	0.0319 (5)	0.0008 (3)	0.0096 (3)	−0.0090 (4)
O3	0.0191 (4)	0.0244 (5)	0.0314 (5)	−0.0025 (3)	0.0008 (3)	0.0130 (4)
C17	0.0150 (5)	0.0134 (4)	0.0139 (4)	0.0011 (3)	0.0021 (3)	−0.0009 (3)
C18	0.0155 (5)	0.0147 (4)	0.0185 (4)	0.0037 (4)	0.0031 (3)	0.0000 (4)
C19	0.0210 (5)	0.0130 (4)	0.0177 (4)	0.0029 (4)	0.0044 (4)	0.0003 (4)
C20	0.0187 (5)	0.0128 (4)	0.0156 (4)	−0.0010 (4)	0.0035 (3)	−0.0006 (4)
C21	0.0150 (5)	0.0147 (4)	0.0183 (4)	0.0008 (4)	0.0030 (3)	0.0006 (4)
C22	0.0161 (5)	0.0118 (4)	0.0181 (4)	0.0018 (3)	0.0032 (3)	0.0004 (4)
N1	0.0177 (4)	0.0157 (4)	0.0134 (4)	−0.0026 (3)	0.0012 (3)	0.0000 (3)
N2	0.0209 (5)	0.0179 (4)	0.0184 (4)	−0.0003 (4)	0.0023 (3)	0.0052 (4)
C1	0.0216 (5)	0.0172 (5)	0.0191 (5)	−0.0032 (4)	0.0004 (4)	0.0039 (4)
C2	0.0241 (6)	0.0150 (5)	0.0253 (5)	−0.0007 (4)	0.0004 (4)	0.0023 (4)
C3	0.0229 (6)	0.0165 (5)	0.0230 (5)	0.0023 (4)	0.0020 (4)	−0.0021 (4)
C4	0.0234 (6)	0.0175 (5)	0.0164 (4)	0.0021 (4)	0.0032 (4)	−0.0001 (4)
C5	0.0159 (5)	0.0156 (4)	0.0138 (4)	−0.0009 (4)	0.0011 (3)	0.0007 (4)
C6	0.0207 (5)	0.0151 (4)	0.0157 (4)	0.0013 (4)	0.0044 (4)	0.0006 (4)
C7	0.0180 (5)	0.0153 (4)	0.0145 (4)	0.0004 (4)	0.0020 (3)	0.0002 (4)
C8	0.0157 (5)	0.0152 (4)	0.0148 (4)	0.0000 (4)	0.0017 (3)	0.0008 (4)
C9	0.0180 (5)	0.0186 (5)	0.0166 (4)	0.0000 (4)	0.0025 (4)	0.0017 (4)
C10	0.0161 (5)	0.0164 (5)	0.0210 (5)	−0.0002 (4)	0.0006 (4)	0.0012 (4)
C11	0.0200 (6)	0.0151 (5)	0.0338 (6)	0.0013 (4)	0.0004 (5)	0.0019 (5)
C12	0.0230 (6)	0.0178 (5)	0.0402 (7)	0.0017 (5)	0.0045 (5)	−0.0072 (5)
C13	0.0269 (7)	0.0238 (6)	0.0290 (6)	0.0002 (5)	0.0073 (5)	−0.0085 (5)
C14	0.0241 (6)	0.0197 (5)	0.0197 (5)	−0.0005 (4)	0.0054 (4)	−0.0030 (4)
C15	0.0154 (5)	0.0152 (4)	0.0173 (4)	−0.0001 (4)	0.0016 (3)	0.0002 (4)
C16	0.0233 (6)	0.0200 (5)	0.0143 (4)	−0.0028 (4)	0.0043 (4)	−0.0022 (4)

### Geometric parameters (Å, °)

**Table:**

Br1—C20	1.8978 (12)	C3—C4	1.3750 (18)
S1—O1	1.4492 (9)	C3—H3A	0.9300
S1—O2	1.4519 (9)	C4—C5	1.4070 (16)
S1—O3	1.4604 (10)	C4—H4A	0.9300
S1—C17	1.7768 (12)	C5—C6	1.4458 (16)
C17—C18	1.3952 (16)	C6—C7	1.3568 (16)
C17—C22	1.3966 (16)	C6—H6A	0.9300
C18—C19	1.3942 (17)	C7—C8	1.4318 (16)
C18—H18A	0.9300	C7—H7A	0.9300
C19—C20	1.3913 (17)	C8—C9	1.3929 (16)
C19—H19A	0.9300	C8—C15	1.4498 (16)
C20—C21	1.3913 (16)	C9—H9A	0.9300
C21—C22	1.3881 (16)	C10—C11	1.3961 (18)
C21—H21A	0.9300	C10—C15	1.4153 (16)
C22—H22A	0.9300	C11—C12	1.386 (2)
N1—C1	1.3599 (16)	C11—H11A	0.9300
N1—C5	1.3712 (15)	C12—C13	1.399 (2)
N1—C16	1.4782 (16)	C12—H12A	0.9300
N2—C9	1.3552 (16)	C13—C14	1.3874 (19)
N2—C10	1.3821 (16)	C13—H13A	0.9300
N2—H1N2	0.850 (19)	C14—C15	1.4079 (17)
C1—C2	1.3669 (19)	C14—H14A	0.9300
C1—H1A	0.9300	C16—H16A	0.9600
C2—C3	1.3971 (19)	C16—H16B	0.9600
C2—H2A	0.9300	C16—H16C	0.9600
			
O1—S1—O2	113.02 (6)	C5—C4—H4A	119.3
O1—S1—O3	112.93 (6)	N1—C5—C4	117.17 (10)
O2—S1—O3	113.24 (6)	N1—C5—C6	118.76 (10)
O1—S1—C17	105.91 (6)	C4—C5—C6	124.07 (10)
O2—S1—C17	106.22 (6)	C7—C6—C5	123.06 (11)
O3—S1—C17	104.63 (5)	C7—C6—H6A	118.5
C18—C17—C22	120.21 (11)	C5—C6—H6A	118.5
C18—C17—S1	121.51 (9)	C6—C7—C8	126.96 (11)
C22—C17—S1	118.27 (8)	C6—C7—H7A	116.5
C19—C18—C17	120.10 (11)	C8—C7—H7A	116.5
C19—C18—H18A	120.0	C9—C8—C7	122.14 (11)
C17—C18—H18A	119.9	C9—C8—C15	105.99 (10)
C20—C19—C18	118.68 (10)	C7—C8—C15	131.86 (10)
C20—C19—H19A	120.7	N2—C9—C8	110.32 (11)
C18—C19—H19A	120.7	N2—C9—H9A	124.8
C21—C20—C19	121.96 (11)	C8—C9—H9A	124.8
C21—C20—Br1	117.86 (9)	N2—C10—C11	128.65 (12)
C19—C20—Br1	120.10 (9)	N2—C10—C15	108.22 (10)
C22—C21—C20	118.82 (11)	C11—C10—C15	123.14 (12)
C22—C21—H21A	120.6	C12—C11—C10	116.88 (12)
C20—C21—H21A	120.6	C12—C11—H11A	121.6
C21—C22—C17	120.21 (10)	C10—C11—H11A	121.6
C21—C22—H22A	119.9	C11—C12—C13	121.35 (12)
C17—C22—H22A	119.9	C11—C12—H12A	119.3
C1—N1—C5	121.55 (10)	C13—C12—H12A	119.3
C1—N1—C16	117.47 (10)	C14—C13—C12	121.62 (13)
C5—N1—C16	120.98 (10)	C14—C13—H13A	119.2
C9—N2—C10	109.17 (10)	C12—C13—H13A	119.2
C9—N2—H1N2	125.1 (13)	C13—C14—C15	118.67 (12)
C10—N2—H1N2	125.5 (13)	C13—C14—H14A	120.7
N1—C1—C2	121.83 (11)	C15—C14—H14A	120.7
N1—C1—H1A	119.1	C14—C15—C10	118.33 (11)
C2—C1—H1A	119.1	C14—C15—C8	135.36 (11)
C1—C2—C3	118.42 (12)	C10—C15—C8	106.29 (10)
C1—C2—H2A	120.8	N1—C16—H16A	109.5
C3—C2—H2A	120.8	N1—C16—H16B	109.5
C4—C3—C2	119.61 (12)	H16A—C16—H16B	109.5
C4—C3—H3A	120.2	N1—C16—H16C	109.5
C2—C3—H3A	120.2	H16A—C16—H16C	109.5
C3—C4—C5	121.40 (11)	H16B—C16—H16C	109.5
C3—C4—H4A	119.3		
			
O1—S1—C17—C18	−98.92 (10)	C3—C4—C5—C6	179.02 (12)
O2—S1—C17—C18	21.50 (11)	N1—C5—C6—C7	177.69 (11)
O3—S1—C17—C18	141.54 (10)	C4—C5—C6—C7	−2.13 (19)
O1—S1—C17—C22	81.05 (10)	C5—C6—C7—C8	−179.49 (11)
O2—S1—C17—C22	−158.53 (9)	C6—C7—C8—C9	−175.13 (12)
O3—S1—C17—C22	−38.49 (10)	C6—C7—C8—C15	3.9 (2)
C22—C17—C18—C19	1.37 (17)	C10—N2—C9—C8	0.64 (14)
S1—C17—C18—C19	−178.65 (9)	C7—C8—C9—N2	178.35 (11)
C17—C18—C19—C20	−0.57 (17)	C15—C8—C9—N2	−0.92 (14)
C18—C19—C20—C21	−0.76 (17)	C9—N2—C10—C11	179.84 (13)
C18—C19—C20—Br1	175.97 (9)	C9—N2—C10—C15	−0.08 (14)
C19—C20—C21—C22	1.26 (17)	N2—C10—C11—C12	−179.37 (13)
Br1—C20—C21—C22	−175.54 (8)	C15—C10—C11—C12	0.53 (19)
C20—C21—C22—C17	−0.44 (17)	C10—C11—C12—C13	0.8 (2)
C18—C17—C22—C21	−0.86 (17)	C11—C12—C13—C14	−1.1 (2)
S1—C17—C22—C21	179.16 (9)	C12—C13—C14—C15	0.1 (2)
C5—N1—C1—C2	−0.78 (18)	C13—C14—C15—C10	1.18 (18)
C16—N1—C1—C2	−179.93 (11)	C13—C14—C15—C8	179.66 (13)
N1—C1—C2—C3	−0.56 (19)	N2—C10—C15—C14	178.40 (11)
C1—C2—C3—C4	1.17 (19)	C11—C10—C15—C14	−1.52 (18)
C2—C3—C4—C5	−0.48 (19)	N2—C10—C15—C8	−0.48 (13)
C1—N1—C5—C4	1.44 (16)	C11—C10—C15—C8	179.60 (12)
C16—N1—C5—C4	−179.43 (11)	C9—C8—C15—C14	−177.76 (14)
C1—N1—C5—C6	−178.40 (11)	C7—C8—C15—C14	3.1 (2)
C16—N1—C5—C6	0.73 (16)	C9—C8—C15—C10	0.84 (13)
C3—C4—C5—N1	−0.81 (18)	C7—C8—C15—C10	−178.33 (12)

### Hydrogen-bond geometry (Å, °)

**Table:**

*D*—H···*A*	*D*—H	H···*A*	*D*···*A*	*D*—H···*A*
N2—H1N2···O2^i^	0.85 (2)	1.91 (2)	2.7593 (14)	175.2 (17)
C1—H1A···O3^ii^	0.93	2.53	3.2067 (16)	130
C7—H7A···O1	0.93	2.58	3.3095 (16)	136
C9—H9A···O1	0.93	2.58	3.2426 (16)	128
C14—H14A···O1^iii^	0.93	2.56	3.2987 (16)	137
C16—H16C···O1^iii^	0.96	2.36	3.2739 (17)	158
C19—H19A···O3^iv^	0.93	2.51	3.2052 (16)	131
C21—H21A···O2^v^	0.93	2.28	3.1310 (15)	152
C4—H4A···Cg3	0.93	2.82	3.5579 (13)	137
C16—H16A···Cg3^vi^	0.96	2.69	3.5731 (13)	154
C16—H16B···Cg1^vii^	0.96	2.74	3.4836 (14)	135

Symmetry codes: (i) −*x*+2, *y*+1/2, −*z*+1/2; (ii) *x*, −*y*+3/2, *z*+1/2;  (iii) −*x*+2, −*y*+2, −*z*+1; (iv) −*x*+2, *y*−1/2, −*z*+1/2; (v) *x*−1, *y*, *z*; (vi) *x*, −*y*+1/2, *z*−1/2; (vii) −*x*+1, −*y*+2, −*z*+1.

## References

[bb1] Allen, F. H., Kennard, O., Watson, D. G., Brammer, L., Orpen, A. G. & Taylor, R. (1987). *J. Chem. Soc. Perkin Trans. 2*, pp. S1–19.

[bb2] Bruker (2005). *APEX2*, *SAINT* and *SADABS* Bruker AXS Inc., Madison, Wisconsin, USA.

[bb3] Chanawanno, K., Chantrapromma, S. & Fun, H.-K. (2008). *Acta Cryst.* E**64**, o1882–o1883. 10.1107/S1600536808027724 PMC2959251 21201095

[bb4] Chantrapromma, S., Laksana, C., Ruanwas, P. & Fun, H.-K. (2008). *Acta Cryst.* E**64**, o574–o575. 10.1107/S1600536808003929 PMC2960744 21201916

[bb5] Chantrapromma, S., Jansrisewangwong, P., Musor, R. & Fun, H.-K. (2009). *Acta Cryst.* E**65**, o217–o218. 10.1107/S1600536808043547 PMC2968146 21581836

[bb6] Chantrapromma, S., Ruanwas, P., Fun, H.-K. & Patil, P. S. (2006). *Acta Cryst.* E**62**, o5494–o5496.

[bb7] Chantrapromma, S., Suwanwong, T. & Fun, H.-K. (2007). *Acta Cryst.* E**63**, o821–o823.

[bb8] Coe, B. J., Harris, J. A., Asselberghs, I., Wostyn, K., Clays, K., Persoons, A., Brunschwig, B. S., Coles, S. J., Gelbrich, T., Light, M. E., Hursthouse, M. B. & Nakatani, K. (2003). *Adv. Funct. Mater* **13**, 347–357.

[bb17] Cosier, J. & Glazer, A. M. (1986). *J. Appl. Cryst.* **19**, 105–107.

[bb9] Dittrich, Ph., Bartlome, R., Montemezzani, G. & Günter, P. (2003). *Appl. Surf. Sci* **220**, 88–95.

[bb10] Jindawong, B., Chantrapromma, S., Fun, H.-K., Yu, X.-L. & Karalai, C. (2005). *Acta Cryst.* E**61**, o1340–o1342.

[bb11] Ogawa, J., Okada, S., Glavcheva, Z. & Nakanishi, H. (2008). *J. Cryst. Growth*, **310**, 836–842.

[bb12] Otero, M., Herranz, M. A., Seoane, C., Martín, N., Garín, J., Orduna, J., Alcalá, R. & Villacampa, B. (2002). *Tetrahedron*, **58**, 7463–7475.

[bb13] Sheldrick, G. M. (2008). *Acta Cryst.* A**64**, 112–122. 10.1107/S0108767307043930 18156677

[bb14] Spek, A. L. (2009). *Acta Cryst.* D**65**, 148–155. 10.1107/S090744490804362X PMC2631630 19171970

[bb15] Weir, C. A. M., Hadizad, T., Beaudin, A. M. R. & Wang, Z.-Y. (2003). *Tetrahedron Lett* **44**, 4697–4700.

[bb16] Yang, Z., Wörle, M., Mutter, L., Jazbinsek, M. & Günter, P. (2007). *Cryst. Growth Des.* **7**, 83–86.

